# Study of the Seawater Desalination Performance by Electrodialysis

**DOI:** 10.3390/membranes12080767

**Published:** 2022-08-05

**Authors:** Jihong Shi, Liang Gong, Tao Zhang, Shuyu Sun

**Affiliations:** 1 Department of Physics, King’s College London, Strand, London WC2R 2LS, UK; 2 Institute of New Energy, China University of Petroleum (East China), Qingdao 266580, China; 3 Computational Transport Phenomena Laboratory (CTPL), Physical Science and Engineering Division (PSE), King Abdullah University of Science and Technology (KAUST), Thuwal 23955-6900, Saudi Arabia

**Keywords:** seawater, desalination, electrodialysis

## Abstract

The global scarcity of freshwater resources has greatly contributed to the development of desalination technologies, in which electrodialysis desalination is one of the most widely used and highly regarded methods. In this work, the first step was to design and assemble a experiment module for electrodialysis desalination. The ion removal efficiency and single membrane mass transfer flux of electrodialysis desalination were investigated. The results show that the desalination performance of the module is improved by increasing the voltage gradient, increasing the concentration of seawater and electrolyte and decreasing the membrane surface flux and that the optimum operating conditions for the module at 24 V operating voltage are feedstock concentration of 35 g/L, electrolyte concentration of 1.42 g/L which and system flow rate of 15 L/h. The results of the study will help to better investigate electrodialysis desalination technology.

## 1. Introduction

With the increasing shortage of freshwater resources worldwide, desalination technology has become a major method of obtaining freshwater in many water-scarce regions [1,2,3]. Currently, desalination technologies can be divided into two main categories: evaporation and thin-membranes methods [4]. Among them, evaporation method has serious scaling and corrosion problems, while the thin film method has become the mainstream method for building plants internationally due to its low energy consumption and high recovery rate [5].

The membrane involving method is mainly divided into two categories: reverse osmosis and electrodialysis. The former has disadvantages, such as high requirements for water quality, low fresh water yield and easy scaling of membranes [6]. As an old desalination method, the latter has the advantages of low requirements for pretreatment, convenient operation and maintenance, less chemical dosage, low energy consumption and long service life of the device and has certain advantages in high brine treatment [7]. Therefore, electrodialysis seawater desalination has become a research hotspot.

Laura [8] designed an electrodialyser in a laboratory consisting of a combination of six negative and seven positive membranes to determine the optimum operating conditions of the electrodialysis system by desalinating brine with a salt concentration of 5 to 10 g/L. Desalination of brine with a salt concentration of 1 to 35 g/L was also conducted, and we found that an operating voltage of 12 V was used to bring the brine solution up to the drinking water standard. We successfully concluded that the desalination function of the electrodialysis system was related to the operating voltage used by the electrodialyser and the salt concentration of the feed.

Mourad [9], in order to further investigate the effect of different operating parameters of the electrodialyser on the desalination effect, used a laboratory electrodialyser, by varying the operating voltage of the electrodialyser, the concentration of the feed brine and the flow rate size of the feed and found that changes in these following factors all had a great effect on the system’s desalination efficiency.

Yoshinobu [10] further investigated the operational performance of the electrodialyser by developing mathematical models for different operations of the electrodialyser and specifically analysed the operational performance of the electrodialyser under continuous, semi-continuous and intermittent operations. The dissociation of water from anion and cation exchange membranes was also investigated, and it was concluded that the metal hydroxides attached to the cation exchange membranes or the functional groups consisting of sulphonic acid groups and quaternary amine groups on the cation exchange membranes can catalyse the dissociation of water and accelerate its dissociation.

As the dissociation of water can lead to scaling of the equipment, the cation exchange membrane needs to be replaced with a new membrane after a certain number of years to reduce the occurrence of dissociation. The question of the cost of desalination in the electrodialysis process was answered by Nikonenko [11] who developed a convection–diffusion model. Using this model, when the operating environment of the electrodialyser is one of high linear flow rates and small membrane spacing, it is economical to reduce costs by reducing the area of the membrane. However, on the other hand, when the membrane performance is superior, a single reduction in membrane area increases the total cost, although energy consumption is reduced; however, the overall operating cost is increased.

Recently, the impact of multivalent ions in seawater on the effectiveness of multistage electrodialysis desalination. Specifically, genuine seawater was employed as the feed solution, and two alternative approaches-using both standard cation exchange membranes (CEMs) and CEMs with selective removal of multivalent ions-were examined. They discovered that the removal of calcium and magnesium was greater for both CEMs compared with the removal of sodium and that operating at low current density had no impact. The multivalent ion permeable CEMs allowed for the removal of more magnesium [12].

Moreover, the removal of the nitrate, arsenic and fluoride by electrodialysis from brackish groundwater were also investigated by Aliaskari et al. [13]. In this study, a batch electrodialysis system was used to explore the effects of operational (flowrate and electricpotential) and water quality (salinity, contaminant feed concentration and pH) factors on brackish water decontamination. However, the composition of the saline groundwater used in the study differed somewhat from that of seawater.

In summary, studies in the literature on electrodialysis desalination have focused on setting up experiments to investigate the factors affecting the desalination process and the operational performance of electrodialysis desalination. However, few studies have discussed the factors influencing desalination performance comprehensively. In this work, the ion removal efficiency and mass transfer flux of the whole module of the desalination process are experimentally calculated, and the major and minor factors affecting the desalination process are quantitatively analysed.

## 2. Experimental Materials

The most important aspect of electrodialysis desalination experiments is the selection of anion and cation exchange membranes as this directly determines the selective transmission of anions and cations in seawater and affects the desalination effect. The commercially available membranes produced by John yong in Neihu District, Taipei, used in this study with the following models: cation exchange membrane (MA-7500) and anion exchange membrane (MC-3475), and the basic information for each commercial membrane is shown in Table 1. The ion exchange membranes used in the system are mainly embedded in the compartment between the electrodialysis modules, and their function is mainly to adsorb anions and cations from the solution to achieve the purpose of purification of the treated water.

The equipment used in the experiment includes a DC power supply, peristaltic electric pump, conductivity meter, pH meter, triple meter, magnet stirrer, balance, deionised water maker, oven, etc. The DC power supply ensures that a stable voltage input is provided to the module, while the experimental brine, deionised water and electrolyte are pumped into the respective chambers via a peristaltic electric pump. The conductivity meter is used to measure the conductivity value of the solution in each chamber before and after the experiment, and the pH meter is used to measure the pH value of the solution in each chamber before and after the experiment. The instrument manufacturers and models are shown in Table 2.

The reagents used in the experiments are shown in Table 3.

## 3. Experimental Platform

**Figure 1:**
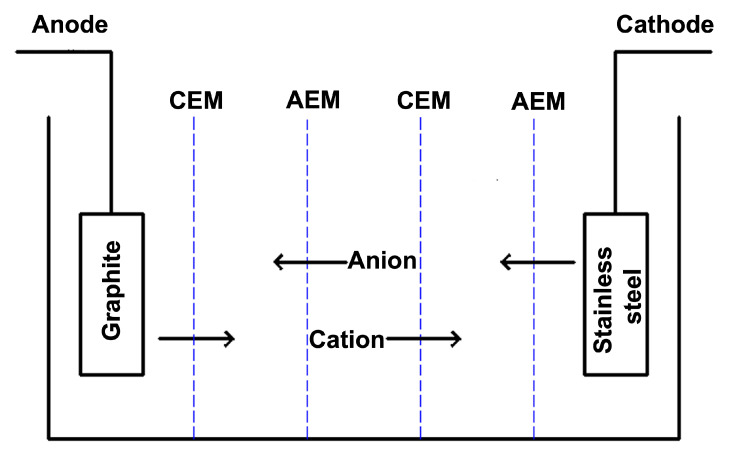
Electrodialysis experiment module.

The electrodialysis experimental module in this study is shown in Figure 1. Inside the module are sequentially arranged anion and cation exchange membranes, which separate the electrodialysis tank into five compartments, with electrode chambers on the left and right sides. The anions and cations in one of the chambers pass through the anion and cation exchange membrane under the action of the power plant and enter the next two chambers, then the conductivity of the solution in that chamber decreases, and the chamber is said to be a low concentration chamber (LCC). The two chambers adjacent to the left and right accept the ions coming from the LCC and prevent them from continuing to migrate into the polar chambers on either side, the ion concentration gradually increases, and these two chambers are called the concentrated chambers.

The raw water is pumped into the LCC by a peristaltic pump and the extracted solution is pumped into the concentrated chamber by a peristaltic pump, where it is sampled and analysed according to the individual residence times, while the electrode chambers on the left and right are steadily fed with electrode solution by another peristaltic pump. The spacer frame of the system is made of an acrylic plate with dimensions of 50 cm long by 30 cm wide by 12 cm thick, the volume of water stored inside the spacer frame is 1600 mL, and the electrode plate used is a graphite plate with dimensions of 50 cm long by 30 cm thick as the anode and a stainless steel plate with dimensions of 50 cm long by 30 cm thick as the cathode. The two electrode chambers are connected in series as a circulation loop, and the treated water from the light electrode chamber is drained.

The entire electrodialysis desalination system is divided into a pair of electrodes and two pairs of anion and cation exchange membranes. The anion and cation exchange membranes are sandwiched between the individual chambers so that each chamber becomes a separate space and finally secured with a C-clamp. The electrode terminals at each end are secured with insulating tape to the two wires which are then connected to the DC power terminals. The anion and cation exchange membrane is cut into small rectangular pieces with an effective area of 25 × 45 cm in size according to the machined dimensions of the acrylic sheet. To simplify the problem under study, NaCl solution was used as the raw water for this experiment.

## 4. Experimental Procedure

Experimental parameters designed as shown below.

Input parameters:

- Fixed factors: simplified model source of seawater, electrodialysis equipment, conductivity measuring instrument and pH measuring instrument.
- Control factors: energising voltage, operating time, flow rate and concentration.

**Figure 2:**
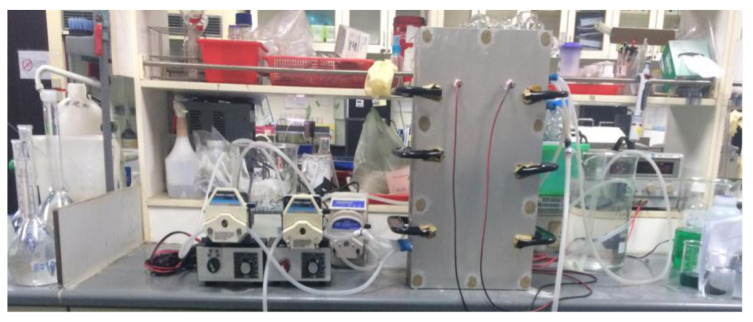
Diagram of the electrodialysis laboratory platform.

Output parameters:

- Ion removal efficiency, pH value of the solution in each chamber and electrical conductivity of the solution in each chamber.

(1) The various parts of the electrodialysis module are connected together to form a desalination system as shown in Figure 2. Before the test is performed, the module needs to be tested for water seal, and if the seal is not good enough, then silicon is used to reinforce it until there is no leakage of any kind.

At the same time, it is also necessary to attempt to run with electricity to see if there is a short circuit in the equipment. If there is a short circuit, it can be overcome by applying grease, white glue and spray paint to the metal nuts or even by abandoning the use of screws for fastening and clamping with C-clamps for fastening of the equipment and by applying silicon to one ring of the equipment so that the equipment finally achieves the requirement of neither leakage nor short circuit.

(2) Prepare 4 L of Na${}_{2}$SO${}_{4}$ solution at a concentration of 0.01 mol/L, which is pumped into the electrode chamber via a peristaltic pump at a flow rate of 15 L/h according to the operational optimum efficiency.

(3) Prepare 6 L of NaCl solution at a concentration of 3.5% and pump it through the peristaltic pump into the light electrode chamber at a flow rate of 15 L/h according to the optimum operating efficiency.

(4) After connecting the power supply box to the equipment with the wire, turn on the power supply and quickly adjust the voltage to the voltage value we need, for this experiment, respectively, for the voltage gradients of 1 and 2 V/cm, i.e., the corresponding voltages are 12 and 24 V, respectively.

(5) Samples were taken every 5 min from the LCC, concentrated and electrode chambers, respectively. Using a conductivity meter and a pH meter, respectively, to measure the corresponding conductivity and pH values and record the corresponding currents.

(6) From the above experimental data, the ion removal efficiency was calculated and analysed and discussed to arrive at the most suitable voltage value, flow rate and removal time for this equipment under certain conditions in order to obtain fresh water.

## 5. Results and Discussion

### 5.1. Effect of Different Voltage Gradients on Ion Removal Efficiency

**Figure 3:**
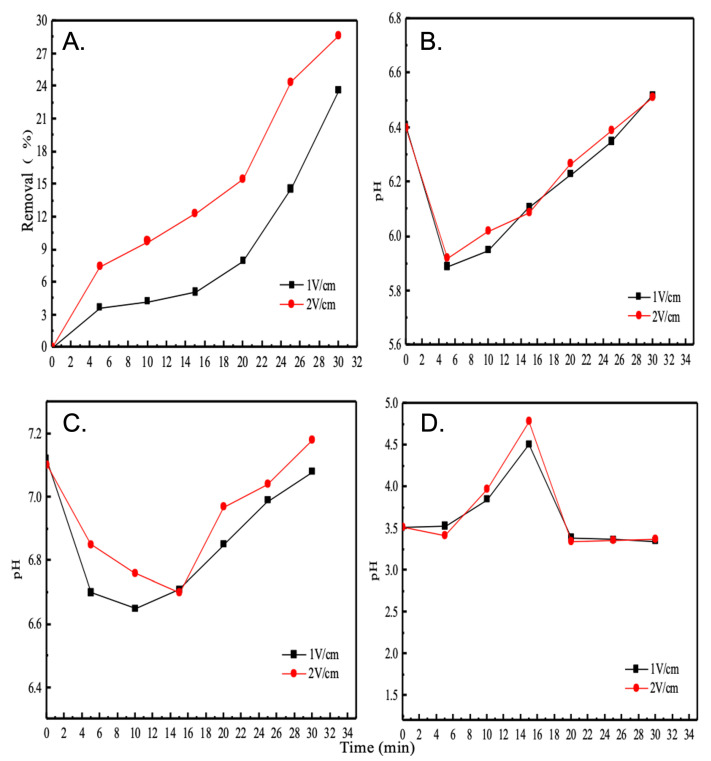
Effect of different voltage gradients on ion removal efficiency (**A**) and variation of pH in each chamber at different voltage gradients (**B**), the concentrated chamber (**C**) and electrode chambers (**D**).

The ion removal efficiency as a function of voltage gradient is shown in Figure 3. As can be seen from the image, the ion removal efficiency increases with increasing voltage gradient and with increasing operating time. The power supply used in this experiment is limited to a maximum voltage of 30 V, i.e., a maximum of 24 V is used to ensure that the experiment provides a voltage gradient of 2 V/cm. Therefore, it is possible that as the voltage gradient increases, the ion removal efficiency will not be increased more effectively. This is because it is possible that the ion diffusion rate on the membranes surface does not accelerate with a higher voltage gradient, resulting in a failure to accelerate the ion removal efficiency. This is a possible scenario with increasing voltage gradients; however, for this set-up, only a voltage gradient of 2 V/cm is discussed.

### 5.2. Variation of pH in Each Chamber at Different Voltage Gradients

The 3.5% brine has a pH of 6.41 when it is not in the electrodialysis system. When it is pumped into the LCC, the pH changes as shown in Figure 3B. 1 V/cm and 2 V/cm voltage gradients make little difference to the pH, which initially drops slightly, and when the working time increases, there is a slight alkalinisation. When deionised water is pumped into the concentrated reaction tank, its pH specifically changes as shown in Figure 3C.

Again, the pH drops slightly at first; however, as the time increases, there is a slight rise in pH of the solution at both voltage gradients, with a slight alkalinisation, and the change curve of pH at a voltage gradient of 2 V/cm is always above 1 V/cm. Furthermore, comparing the concentrated chamber with the LCC, it can be seen numerically that the degree of alkalinisation is higher in the concentrated chamber than in the LCC.

The reason for the micro-alkalisation may be due to the hydrolysis reaction on the surface of the membrane, especially as the ion concentration in the brine decreases, the water molecules are easily decomposed of polarisation, producing H${}^{+}$ or OH${}^{-}$ on the different membrane surfaces, thus, causing the micro-alkalisation phenomenon. It is important to note that the pH value of the brine is still in the neutral range after treatment, which has a positive technical value.

The change in pH within the two electrode chambers, as shown in Figure 3D, at the beginning, the pH of the aqueous solution will gradually increase, which is due to the decomposition of water molecules on the surface of the electrode process H${}^{+}$ or OH${}^{-}$, with the extension of time, the reaction of the long born H${}^{+}$ is more intense, the pH will gradually decrease, when the role to about 20 min—that is, to reach a stable state.

### 5.3. Variation of the Conductivity of the Chambers at Different Voltage Gradients

**Figure 4:**
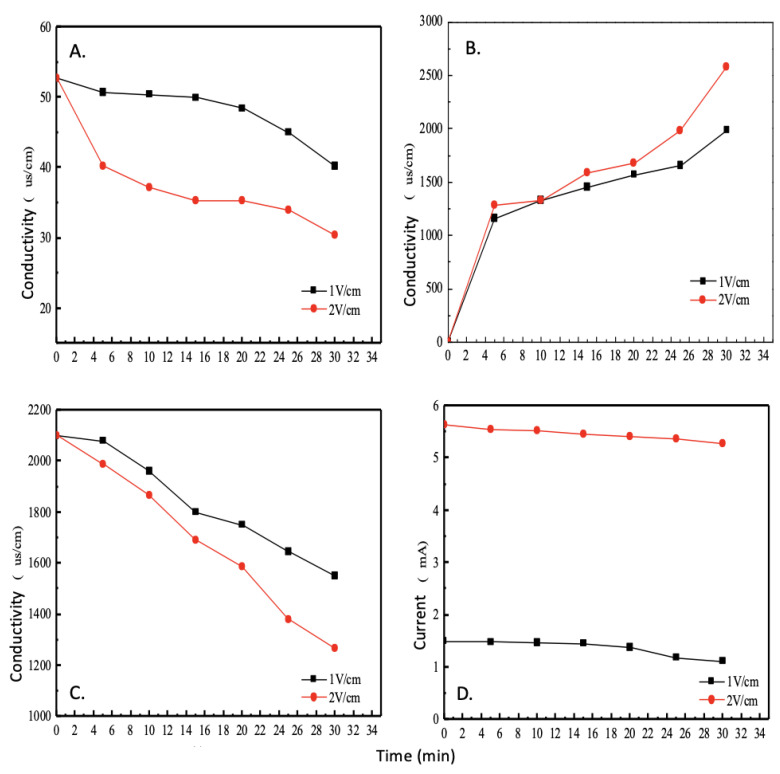
Variation of the conductivity of the chambers at different voltage gradients ((**A**) LCC), the concentrated chamber (**B**), electrode chambers (**C**) and current consumption at different voltage gradients (**D**).

A higher conductivity value indicates a higher concentration of metal ions within the solution and can therefore be used to quantify the ion removal efficiency of this electrodialysis module. 3.5% brine has an initial conductivity value of 52.7 mS/cm. As shown in Figure 4A, the solution in the LCC shows a significant decrease in conductivity as the working time increases.

Conversely, as shown in Figure 4B, the solution in the concentrated chamber showed a significant increase in conductivity as the working time increased, and the larger the voltage gradient, the greater the amount of change in conductivity. Furthermore, at a voltage gradient of 2 V/cm, the electrodialysis module needs to work for approximately 3 h before the conductivity values within the LCC are between 200 and 350 μs/cm, while the conductivity of normal tap water, between 200 and 500 μs/cm [14], are comparable.

The solution inside the electrode chamber, as shown in Figure 4C, shows a gradual decrease in conductivity as the solution is continuously reacted and the number of ions inside slowly decreases as the electrolysis progresses. Furthermore, at a voltage gradient of 2 V/cm, the conductivity inside the electrode chamber decreases faster than at 1 V/cm, and the slope of the curve is larger.

### 5.4. Current Consumption at Different Voltage Gradients

As shown in Figure 4D, at different voltage gradients, the current change of the electrodialysis system shows that the initial current is proportional to the voltage gradient—that is, the larger the voltage gradient causes the faster the migration of free electrons in the solution, as the faster the migration of electrons can generate a larger current. Thus, with the increase of the voltage gradient, a higher removal efficiency can be achieved, in addition, as the working time increases, the solution becomes less free electrons, the movement of ions will become slower, so the current will decrease with the reduction of the system conductivity value.

### 5.5. Effect of Seawater Concentration on Mass Transfer Fluxes

**Figure 5:**
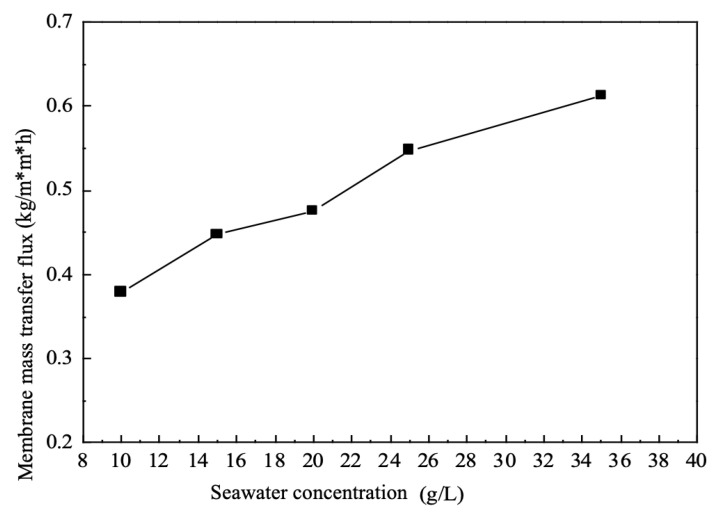
Effect of seawater concentration on mass transfer fluxes.

As can be seen from Figure 5, the single membrane mass transfer flux gradually increases as the brine concentration increases, all other conditions being held constant. This is because in this group of electrodialysis equipment, deionised water is used as the drawing fluid, which ensures that the brine The concentration of the brine will always be much higher than the concentration of the draw solution, and the anions and cations in the brine will migrate towards the concentration chambers on either side of the brine due to the difference in concentration potential and potential potential difference.

The concentration difference between the brine and the draw solution is the driving force for ion migration. In the case of a certain voltage gradient and draw solution concentration, gradually increasing the concentration of the brine will make the concentration difference between the raw material and the draw solution larger, and the driving force between the two will also become larger; at the same time, as the brine concentration increases, the resistance of the electrodialysis system also becomes smaller, i.e., the mass transfer resistance becomes smaller.

The ratio of the driving force to the system resistance determines the mass transfer rate of the electrodialysis system, and thus the driving force is increasing while the resistance is decreasing, and under the joint action of the two, the whole system shows an increase in the mass transfer rate and an increase in the mass transfer flux.

### 5.6. Effect of Flow on Mass Transfer Fluxes

**Figure 6:**
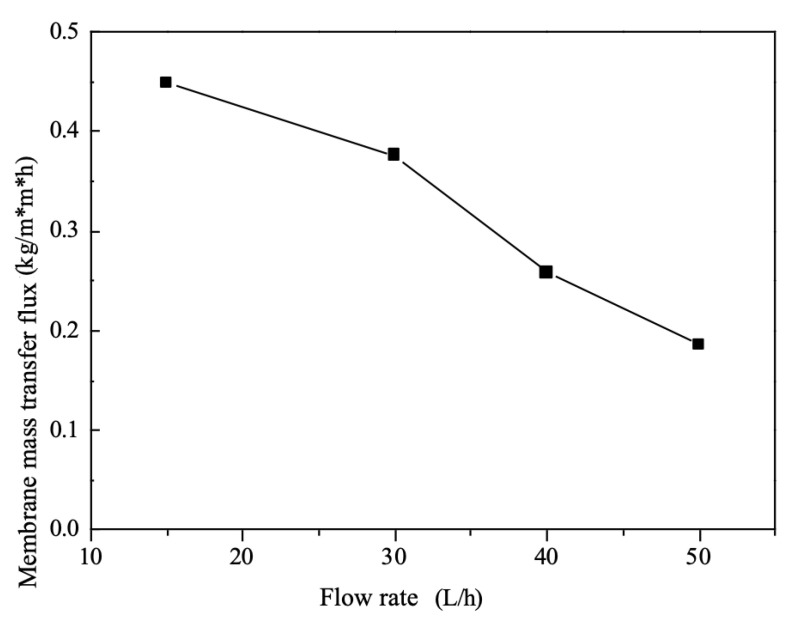
Effect of the flow rate on mass transfer fluxes.

The mass transfer flux of the system decreases when the flow rates of the feed and draw liquids increase simultaneously, as shown in Figure 6, such a conclusion is consistent with the results of Sadrzadeh M [15,16].The total mass transfer resistance of the electrodialysis system is concentrated within the diffusive boundary layer. As the flow rate of the brine increases, the perturbation of the flow slug also increases within the freshwater chamber, i.e., the degree of fluid turbulence increases, which causes the thickness of the diffusion boundary layer to decrease, then the diffusion of ions from the solution to the boundary layer of the brine increases, then the solution resistance on the brine side decreases.

However, at the same time, as the flow rate of the draw fluid increases, the solution resistance within the concentration chamber on both sides will increase with the flow rate The overall total resistance of the system increases with flow rate. On the other hand, the transfer process of ions in the membrane is essentially an alternating process of selective adsorption and desorption [17], and an increase in flow rate decreases the time the solution stays on the surface of the ion exchange membrane.

### 5.7. Effect of Electrolyte Concentration on Mass Transfer Fluxes

**Figure 7:**
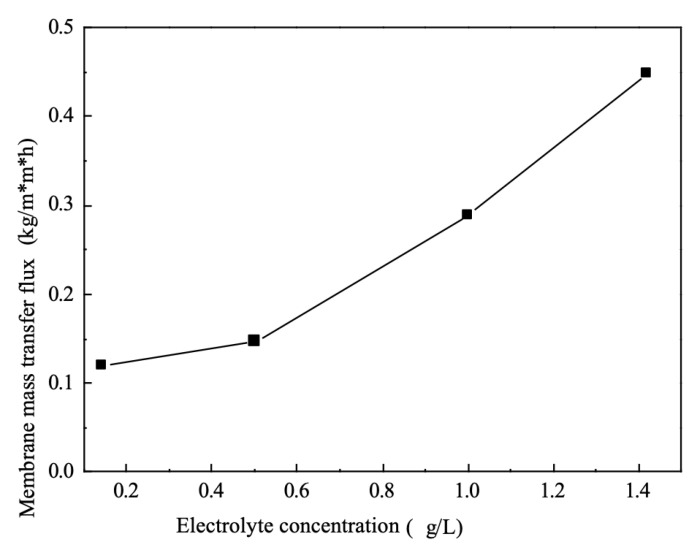
Effect of electrolyte concentration on mass transfer fluxes.

As can be seen from Figure 7, as the concentration of the electrolyte increases, the single membrane mass transfer flux increases throughout the electrodialysis system. This is due to the fact that the current in the electrodialysis unit is made up of two parts; the conductivity in this part of the external power supply at both extremes is achieved by the directional movement of electrons, while the conductivity between the solutions located within the module is achieved by the migration of ions. This means that throughout the circuit of the electrodialysis module there must be a transition from electronic to ionic conductivity, or ionic to electronic conductivity.

This part of the bridge-like conductive articulation is dependent on a redox reaction occurring within the electrode chambers at both ends [18]. The ions in the electrolyte act as the medium for this transition. When the concentration in the electrolyte is low, polarisation may occur, leading to scaling of the electrode plates within the chamber, which can increase energy consumption during desalination and reduce the life of the plates. When the concentration of the electrolyte increases, the number of ions increases, which increases the strength of electron conductivity into solution ion conductivity.

At the same time, as the concentration of the electrolyte increases, the resistance of the electrode zone decreases with the same operating voltage, which is shown to increase the mass transfer flux of the membrane.

## 6. Conclusions

In this paper, an electrodialysis desalination experimental module was constructed, the major and minor factors affecting the desalination performance of electrodialysis were discussed, and the following conclusions were obtained. Through experimental verification, we found that the most critical factor affecting desalination performance in the electrodialysis module was the magnitude of the voltage gradient, with the next most influential factors being the feedstock concentration, flow rate and electrolyte concentration, in that order.

Increasing the voltage gradient, feedstock concentration and electrolyte concentration of the electrodialysis desalination system and reducing the flow rate of the system will result in a higher desalination performance of electrodialysis, thus, making the electrodialysis desalination process more energy efficient. For this electrodialysis desalination module, the optimal working conditions are an operating voltage of 24 V, raw material concentration of 35 g/L, electrolyte concentration of 1.42 g/L and flow rate of the system of 15 L/h.

## Figures and Tables

**Table 1:** The basic information for commercial membranes used in this work.

Items	MA-7500	MC-3475
Total weight (oz./sq.yd.)	11.43	3.46
Membrane thickness (mm)	0.53	0.38
Capacity (meq./g.)	1.116	0.70
Water permeability (cc/hr./ft@5psig)	4.49	30
Chemical Stability (pH)	1∼10	1∼10
Ionic Form	Sodium	Chloride

**Table 2:** Manufacturers and models of the laboratory equipment used.

Instrument Name	Manufacturer	Instrument Model
DC Power Supply	Jehan Technology Ltd	GPR-3060D
Peristaltic Electric Pump	Cole-Parmer	7524-40
Conductivity Meter	Suntex	SP-700
pH Meter	Suntex	SP-701
Triple Use Electricity Meter	TES	TES-2801
Magnet Stirrer	Corning	CP-420
Balance	Precisa	3100C
Balance	AND	ER-120A
Deionized Water Maker	Millipore	SA-67120
Oven	Deng Yong	DS-45

**Table 3:** Reagents used in the experiment.

Name of Reagent	Manufacturer	Remarks
Sodium chloride	Shimakyu Pharmaceutical Co.	Analytical purity
Sodium sulfate anhydrous	EMD Millipore Corporation, Germany	Analytical purity
Deionized water	Laboratory	Analytical purity

## Data Availability

Data will be made available on request.
